# Supplementary material for: Molecular profiling of the artemisinin resistance Kelch 13 gene in Plasmodium falciparum from Nigeria
Source: PLoS One. 2022 Feb 28;17(2):e0264548. doi: 10.1371/journal.pone.0264548 (PMC8884509; doi:10.1371/journal.pone.0264548)
Supplement: Attachment — Submitted filename: Review report PONE-D-21-25383R1.pdf [file pone.0264548.s002.pdf]

Comments/suggested changes from the editor:

General:

Please use abbreviations consistently throughout the manuscript and write them in full the first time they appear in the manuscript. There are also a couple of typos and some grammar errors which should be corrected.

Title:

1. Change to "Molecular profiling of the artemisinin resistance Kelch 13 gene in *Plasmodium falciparum* from Nigeria"

Introduction:

2. Line 57: Delete "The" before "Artemisinin-resistant".
3. Line 59: Also cite doi: 10.1056/NEJMc0805011 alongside REF #3
4. Line 60: The first sentence should be deleted.
5. Line 64: Cite REF #8 alongside REFs #11 and #12.
6. Lines 64-65: "Due to these mutations, the efficacy of ACTs may be compromised". REF #13 is inappropriate. Furthermore, I would expect some lines about partner drug resistance here. See also comment of Reviewer #1.
7. Line 69: Delete REF #15 (Colombian study).

Materials and Methods:

8. Line 141: iii) Asexual parasite clearance time (PCT) to line 142. I also suggest including the commonly used terms ACPR, ETF, LCF, and LPF, respectively, as used in the final TES report. These would not have to be described/defined in detail in the manuscript; the official WHO TES study protocol can be cited instead.
9. Lines 145-146: This sentence needs to be reworded.

Results:

10. Between "Demographics" and *Pfk13* gene mutations", a short summary of the TES results (i.e., summary table of crude and PCR-corrected ACPR, ETF, LCF, and LPF) of the 300 samples would be very helpful.
11. Lines 222-224: Replace with: "Thirteen pfk13 gene mutations were detected in 21 out of the 332 sequences analyzed in this study (Table 2)."
12. Line 225: Delete "and".
13. In Figure 1 and Table 3, please order the SNPs in ascending order according to the location in the gene.
14. Lines 226 and 227: Replace "sequences" with "samples".
15. Table 3 was referred to in line 224. However, this is not correct because the text outlined the results of the current study. Table 3 summarises the results of all Nigerian studies hitherto conducted; this should be mentioned in the text. On another note: Is there a specific reason why

the polymorphisms reported by Abubakar *et al*. (2020; DOI: 10.3390/tropicalmed5020085) are not included in Table 3?

16. Line 251: "…(mean time to recurrence: 29±6.3 days)."
17. Footnote Table 4/Line 263: "…recurrent parasitaemia"
18. Lines 266-267: Replace with "Comparison of responsiveness indices following treatment initiation in children with and without mutated *Pfk13*."

Discussion:

19. The first sentence of the Discussion should be toned down, particularly in view of the fact that no SNPs associated with artemisinin resistance were observed and nothing is reported on partner drug resistance.
20. Line 300: Replace "is" with "was".
21. Line 317: Replace "has" with "have".
22. Line 322: "…are less likely to be associated with a delayed parasite clearance phenotype…"
23. Lines 324-326: "In addition, significantly longer asexual parasite clearance times in children infected with non-mutant *Pfk*13 parasites indicate that mutants identified in the parasites circulating in Nigeria do not confer resistance to artemisinin derivatives.

Supporting Information:

24. The Supporting Information could be omitted. Instead, a reference to the WHO protocol for parasite genotyping to differentiate recrudescence from new infections could be in included in the footnote of the summary table I suggested in comment # 10.
