# Supplementary material for: Molecular profiling of the artemisinin resistance Kelch 13 gene in Plasmodium falciparum from Nigeria
Source: PLoS One. 2022 Feb 28;17(2):e0264548. doi: 10.1371/journal.pone.0264548 (PMC8884509; doi:10.1371/journal.pone.0264548)
Supplement: Attachment — Submitted filename: Response to Reviewers.docx [file pone.0264548.s001.docx]

Review Comments to the Author

**Reviewer #1**

Artemisinin resistance is a serious threat to malaria control and elimination, and monitoring of antimalarial drug efficacy and resistance is crucial to help policymakers make informed decisions. The authors present comprehensive data on the prevalence of K13 mutations in different states of Nigeria, and try to understand if the mutations found may have an impact on the efficacy of artemisinin- based combination therapies. However their manuscript has several weaknesses. First the background information is outdated. It is surprising that the authors mention only India when discussing the spread of that artemisinin resistance outside the Greater Mekong Sub-region. There are reports of validated K13 mutations in South America, but more importantly there are reports of validated K13 mutations in Rwanda associated with delayed parasite clearance. I think these two publications should be cited : PMID32747827, PMID33864801, and the local emergence of artemisinin resistance in Sub Saharan Africa discussed.

The second major limitation of the study is the use of K13 sequences to construct phylogenetic trees. Indeed K13 is under drug pressure and is not the right marker to assess genetic diversity especially in samples collected from a clinical trial. I would recommend the authors to remove the data from the manuscript as they may be misleading. The second comment on the methodology section is the assessment in silico assessment of protein-ligand docking. This manuscript should focus on the monitoring of known and new mutations in K13. The provide information on protein structure is not useful here. There are already many validated or candidate K13 mutations, and the studies looking at the protein-ligand should focus on these mutations. The authors should maybe have used resources to assess molecular markers associated with partner drug resistance, or to establish ex vivo assay to look for new potential markers.

**Response:** We appreciate the reviewer for your comments. We have now significantly improved the manuscript and addressed the weaknesses you pointed out as shown in our responses to your specific comments.

**Specific comments**

- Introduction, line 58: Artemisinin derivatives were not used in 168 BC, artemisinin was isolated in 1972. In traditional Chinese medicine, Artemisia annua plants were prepared with hot water to treat fever, and this cannot probably lead to resistance

**Response:** We appreciate the reviewer for this comment. We have now rephrased the introduction. Line 51 - 67 in the revised manuscript now reads …

“Malaria, one of the major global health challenges, has co-existed with humans for over 40 centuries. Control of this ancient disease heavily relies upon the use of antimalarial drugs [1]. Artemisinin-based combination therapies (ACTs) [e.g., artemether-lumefantrine (AL) and artesunate-amodiaquine (AA)] are the current line of treatment for malaria. These drugs were recommended by the World Health Organization (WHO) as first-line treatment for uncomplicated falciparum malaria in 2001 and have since been widely adopted to treat falciparum malaria globally [2]. The Artemisinin (ART)-resistant *P. falciparum* has been confirmed to have emerged from the Greater Mekong Subregion (GMS) Thai-Cambodian border [3] and has spread to other malaria endemic regions [4 – 7].

The *pfk13* gene has been reported to be associated with ART-resistance [8, 9]. Several point mutations in the *pfk13* gene (F446I, N458Y, M476I, Y493H, R539T, I543T, P553L, R561H, P574L and C580Y) have been validated to correlate with clinical ART resistance in Southeast Asia and South America [2, 10] and confirmed to confer elevated survival rates based on Ring-stage Survival Assays (RSA)0–3 h [11, 12]*.* Due to these mutations, the efficacy of ACTs may be compromised [13]. Recently for the first time in Africa, two of the validated mutations (R561H and P574L) on the *pfk13* gene have been reported in Rwanda to be associated with *in vitro* resistance to ACTs [6, 7].”

- Introduction, Line 61: The list of validated and candidate mutations in K13 has been continuously updated. please refer to the last WHO report: <https://www.who.int/publications/i/item/9789240012813>

**Response:** We appreciate the reviewer for this comment. This statement has been updated in the manuscript. Line 60 - 63 in the revised manuscript now reads …

“The *pfk13* gene has been reported to be associated with ART-resistance (8, 9). Several point mutations in the *pfk13* gene (F446I, N458Y, M476I, Y493H, R539T, I543T, P553L, R561H, P574L and C580Y) have been validated to correlate with clinical ART resistance in Southeast Asia and South America (2,10) …”

- Introduction, lines 63-65: K13 mutations are associated with delayed parasite clearance, not full resistance. When there is no resistance to partner drug, patients are still cured even with delayed parasite clearance.

**Response:** We appreciate the reviewer for this comment. This statement has now been rephrased in the manuscript. Line 60 - 67 in the revised manuscript now reads …

“The *pfk13* gene has been reported to be associated with ART-resistance [8, 9]. Several point mutations in the *pfk13* gene (F446I, N458Y, M476I, Y493H, R539T, I543T, P553L, R561H, P574L and C580Y) have been validated to correlate with clinical ART resistance in Southeast Asia and South America [2, 10] and confirmed to confer elevated survival rates based on Ring-stage Survival Assays (RSA)0–3 h [11, 12]*.* Due to these mutations, the efficacy of ACTs may be compromised [13]. Recently for the first time in Africa, two of the validated mutations (R561H and P574L) on the *pfk13* gene have been reported in Rwanda to be associated with *in vitro* resistance to ACTs [6, 7].”

- Introduction, lines 65-66: the statement is incorrect. As mentioned in my general comment, delayed parasite clearance was associated with a validated marker (R561H) in Rwanda: PMID33864801

**Response:** We appreciate the reviewer for this comment. As mentioned in our previous response, this statement has now been rephrased.

- Material and Methods

Could you please give a brief description of the study sites or provide a reference to the original study

**Response:** We appreciate the reviewer for this comment. The link to the original report of the study sites has been provided in the manuscript draft. Line 102 - 103 in the revised manuscript now reads …

“…Full description of study sites is available online at https://www.health.gov.ng/doc/2018-TES-FINAL-REPORT.pdf.”

- Enrollment and sample collection

Could you please mention which drugs were assessed in the original study

**Response:** We appreciate the reviewer for this comment. However, this information was previously provided in the manuscript. Line 95 - 98 in the manuscript reads …

“This is a retrospective, cross-sectional, community-based study which is part of the therapeutic efficacy study (TES) for monitoring antimalarial efficacies of Artesunate-amodiaquine (AA), Artemether-lumefantrine (AL) and Dihydroartemisinin-piperaquine (DHP) in the treatment of uncomplicated *P. falciparum* infections in children aged 6-96 months old in Nigeria.”

- Results, demographics

The geometric mean of parasitemia is 17'765 parasites /ul, however, in the inclusion criteria, the minimum parasitemia is 20'000 parasites/ul

**Response:** We appreciate the reviewer for this comment. However, the minimum parasitemia stated in the original manuscript draft is 2000 parasites/ul. Line 109 - 113 in the manuscript reads …

“Children were enrolled if they were 6-96 months old. Children were eligible for enrolment if they had symptoms compatible with acute uncomplicated malaria, *P. falciparum* mono-infection with parasite count ranging from 2,000 - 200,000 asexual forms/μl by microscopy and body (axillary) temperature of ≥ 37.5 °C or a history of fever in the 24 hours preceding presentation.”

- Table 1

Could you please clarify for which states, the different variables are statistically significant

**Response:** We appreciate the reviewer for this comment. However, this was previously mentioned in the manuscript. Line 212 - 214 in the manuscript reads ...

“Children enrolled in Kano State were significantly (p<0.0001) younger and had significantly (p=0.046) lower enrollment asexual parasitaemia compared to children enrolled in other States (Table 1).”

- PfK13 gene mutations and table 2

Please specify which mutations are synonymous and non-synonymous

**Response:** We appreciate the reviewer for this comment. We have now updated Table 2 in the revised manuscript to indicate synonymous and non-synonymous mutations.

**Table 2. *Pfk13* polymorphisms observed in Kano, Enugu and Plateau States**

| **S/N** | **K13 Amino Acid Locus** | **Nucleotide Locus** | **Reference Allele** | **Mutant Allele** | **Mutation Type** | **Country Previously Observed** | **Nigerian State Observed** |
| --- | --- | --- | --- | --- | --- | --- | --- |
| 1. | K438N | 1314 | A | T | Non-synonymous | SE Asian Countries | Kano |
| 2. | G449S | 1345 | G | A | Non-synonymous | Mali | Kano |
| 3. | F451L | 1353 | T | - | Non-synonymous | - | Kano |
| 4. | C469C | 1407 | C | T | Synonymous | Kenya, Malawi,  Senegal,  Niger, Congo, DRC | Enugu |
| 5. | V487E | 1460 | T | A | Non-synonymous | - | Enugu |
| 6. | A557S | 1669 | G | T | Non-synonymous | Congo, DRC, Côte d’Ivoire | Enugu |
| 7. | A578S | 1732 | G | T | Non-synonymous | Uganda,  Kenya, DRC, Gabon, Mali, Ghana, Cameroon, Cambodia,  India | Enugu |
| 8. | Q613H | 1839 | A | T | Non-synonymous | Senegal, Ghana, Tanzania | Enugu & Plateau |
| 9. | A621A | 1863 | T | A | Synonymous | - | Enugu |
| 10. | Q661H | 1983 | A | - | Non-synonymous | - | Enugu |
| 11. | N664I | 1991 | A | T | Non-synonymous | - | Plateau |
| 12. | V692G | 2075 | T | G | Non-synonymous | - | Enugu & Plateau |
| 13. | N694K | 2082 | T | A | Non-synonymous | Angola, Cote d’Ivoire | Kano |

- Table 4

Could you please clarify in table 4 if PPT was recrudescence or new infection.

**Response:** We appreciate the reviewer for this comment. Table 4 has now been updated in the revised manuscript.

**Table 4: Clinical features of children with mutated *P falciparum* in this study.**

| Study site | Sample ID | Gender | Age (Month) | Enrollment asexual parasitaemia (uL^-1^) | Mutation Position | Parasite clearance time (day) | Antimalarial Treatment | Treatment outcome |
| --- | --- | --- | --- | --- | --- | --- | --- | --- |
| Enugu | 33 | M | 48 | 30571 | A578S | 1 | AL | ACPR |
|  | 89 | F | 60 | 12817 | Q613H | 2 | AL | ACPR |
|  | 106 | F | 36 | 16290 | Q613H | 2 | AL | ACPR |
|  | 134 | M | 72 | 57850 | A621A | 2 | AL | ACPR |
|  | 154 | M | 60 | 3424 | V487E | 2 | AL | ACPR |
|  | 179 | M | 36 | 61320 | A557S | 2 | DHP | ACPR |
|  | 197 | M | 48 | 2055 | C469C | 2 | DHP | PPT  (D42) |
|  | 228^+^ | F | 24 | 91680 | C469C | 2 | AL | PPT  (D21) |
|  | 236 | M | 36 | 9794 | C469C | 2 | AL | PPT  (D28) |
|  | 239 | M | 72 | 6714 | V692G  Q661H | 1 | AL | PPT  (D28) |
|  | 254 | M | 96 | 29229 | C469C | 2 | AL | PPT  (D28) |
| Kano |  |  |  |  |  |  |  |  |
|  | 14 | M | 12 | 15923 | N694K | 2 | AA | ACPR |
|  | 26 | F | 24 | 37880 | F451L | 1 | AL | ACPR |
|  | 51 | F | 84 | 26523 | K438N | 2 | AA | ACPR |
|  | 59 | M | 36 | 16888 | G449S | 2 | AL | ACPR |
|  | 77 | M | 48 | 2250 | G449S | 1 | AA | ACPR |
|  |  |  |  |  |  |  |  |  |
| Plateau | 31 | F | 84 | 26069 | Q613H | 1 | AA | ACPR |
|  | 58 | M | 60 | 16304 | Q613H | 1 | AL | ACPR |
|  | 62 | F | 72 | 61080 | Q613H | 2 | AL | ACPR |
|  | 77 | F | 60 | 5428 | N664I | 1 | AL | PPT  (D28) |
|  | 155 | M | 60 | 54739 | V692G | 2 | AA | PPT  (D28) |
| Mean (SD) |  |  | 53.71  (22) | 17, 530* |  | 1.67 (0.48) |  | 29  (6.3)^#^ |

***Geometric mean; ^#^Mean PPT: Parasite post treatment, AA: Artesunate-amodiaquine; AL: Artemether-lumefantrine; DHP: Dihydroartemisinin-piperaquine, ACPR: Adequate clinical and parasitological response**

**^+^Only sample with recrudescence parasitaemia**

- Discussion

Lines 355-357: The statement is confusing. I guess you mean that the codon 578 is close to 580. But it does not mean that the former mutation is important because it is close to a mutation that is strongly associated with parasite clearance. Several studies have shown that this mutation is not associated with delayed parasite clearance. Whereas its monitoring is important due to its relatively high frequency in sub Saharan Africa, you should not overestimate its importance.

**Response:** We appreciate the reviewer for this comment. We have now adjusted this statement in the revised version of the manuscript. Line 306 - 315 in the revised manuscript now reads …

“In addition, eight (A578S, C469C, Q613H, K438N, A621A, N694K, G499S and A557S) of the 13 SNPs observed in our study have also been observed in Southeast Asia and other sub-Saharan African countries [8, 20, 21, 24, 26, 27, 29, 32, 33, 37 – 41] as represented in Table 3. The Q613H was the most prominent SNP observed in our study and was also reported in recent studies in Nigeria [22, 23]. A578S is, however the most prominent SNP observed among sub-Saharan African countries such as Ghana, Kenya, Gabon, DRC, Uganda, Cameroon and Mali [27, 32, 40]. This mutation (A578S) represents a change from a non-polar to a polar amino acid and can alter the shape of the *pfk13* protein in the regions it has been observed in [28]. Therefore, more attention must be paid to this mutation as it is emerging to be the most prominent mutation observed in the *pfk13* gene in sub-Saharan Africa [27, 32, 40].”

- Lines 389-391: Your phylogenetic analysis is concluding that you parasites are clustering with parasites from India, Kenya and Uganda. However when looking at table 2, only the widespread A578S was also found in those countries, and not the other mutations found in your studies, except the synonymous mutation (469). How could those mutations be introduced from countries where they did not exist ? Again, this is probably due to the limitation of the marker you used to construct your phylogenetic analysis.

**Response:** We appreciate the reviewer for this comment. We have now removed the phylogenetic analysis from the manuscript.

**Reviewer #2**

This is a molecular survey for alleles in Kelch-13 gene of P. falciparum and not designed to describe the potential emergence and spread of ART resistance. The authors noted that resistance could be via other mechanisms. For a country with over 30 geopolitical regions, the sample is hardly representative of the population of Nigeria. Malaria transmission is overall high in Nigeria but heterogeneous and this could affect the level of drug pressure across the country, allowing for different patterns of evolution of known and emerging drug resistance associated with genetic loci. Overall, the study is important as any resistance to artemisinins in Nigeria will seriously hamper hopes of elimination of the disease in Africa.

Major issues

- Assent was only for participants at 84-96 months. What of younger children?

**Response:** We appreciate the reviewer for this comment. However, for such children, informed consent was obtained from parents or legal guardians.

- Phylogenetic trees are based on mutation rates and not an accurate representation for a recombinant organism such as P. falciparum. A neighbour-joining tree based on genetic distances would be more appropriate.

**Response:** We appreciate the reviewer for this comment. We have removed the phylogenetic analyses from the manuscript based on your recommendation and that of the other reviewers.

- BEAST is not optimised for recombination seen in eukaryotic organisms such as P. falciparum and this is a major issue with drawing trees. Several mutation models were determined with the same data and these are not accurate due to the absence of a recombination graph.

**Response:** We appreciate the reviewer for this comment. We have removed BEAST analysis from the manuscript based on your recommendation and that of the other reviewers.

- What was the rationale for protein ligand docking, as ART does not directly bind to pfk13. This gives the impression of scouting for data rather than a hypothesis orient investigation or a simple survey as applied in this case. The result of docking rather describes the methods. This section is irrelevant and adds nothing to the data.

**Response:** We appreciate the reviewer for this comment. We have now removed the protein-ligand docking analysis from the manuscript.

- The highest frequency mutation was less than 2% (not more than 6 individuals overall). In table 4, only C469C was reported in more than 2 individuals. Interestingly this is a synonymous variant but seemed to be occurring in individuals with presence of parasitaemia from 3 weeks post commencement of treatment. However, it is hard to consider this number enough to statistically classify the age distribution or correlation with treatment outcome. Being mostly from Enugu, could this be a clonal outbreak carrying the mutation and causing re-infections. There was no indication if these post treatment samples only or were present prior to commencement of treatment

**Response:** Thank you for your thoughts on the clonal outbreak which could be the case however, we did not conduct a high resolution experiment to investigate this possibility. Also, the C469C SNP was only present in post treatment samples from Enugu.

Minor Issues

- Introduction

Line 57: The authors stated that ART monotherapy use in China drives drug pressure, but that did not account for the fact that ART-resistant parasites first evolved along the Thai Cambodia border, which is downward on the southern part of the Greater Mekong Sub-region. Perhaps, the escape and spread of ART-resistant isolates from China along the Thai-Cambodia border could have increased the rate of fixation of ART-resistant parasites in that region.

**Response:** We appreciate the reviewer for this comment. We have now rephrased the introduction.

- Line 62: Please rephrase, it is the survival rate that is elevated n

**Response:** We appreciate the reviewer for this comment. We have rephrased this statement in the manuscript. Line 60 - 64 in the revised manuscript now reads …

“The *pfk13* gene has been reported to be associated with ART-resistance [8, 9]. Several point mutations in the *pfk13* gene (F446I, N458Y, M476I, Y493H, R539T, I543T, P553L, R561H, P574L and C580Y) have been validated to correlate with clinical ART resistance in Southeast Asia and South America [2, 10] and confirmed to confer elevated survival rates based on Ring-stage Survival Assays (RSA)0–3 h [11, 12]*.*”

- Discussion:

The paragraph on protein-ligand docking (starting from line 371) should be combined or commenced with a linking word which contrasts the immediate previous paragraph. This is because even though the amino acid substitutions observed have a destabilising effect on the protein structure, they do not necessarily affect drug efficacy.

**Response:** We appreciate the reviewer for this comment. We have now removed the protein-ligand docking analysis and corresponding discussions from the manuscript.

**Reviewer #3**

This article by Ajogbasile et al, reports the results of a molecular surveillance of the pfk13 gene conducted in Nigeria in 2018. Previously, some mutations in the propeller part of this gene have been described as correlated with artemisinin resistance in South East Asia, in the Guiana Shield and in some African countries as Rwanda and Uganda. 332 samples have been analyzed in Nigeria and 13 pfk13 mutations identified in the propeller part of the gene. The manuscript is interesting but could be improved if it clearly mentions and associates the TES. The sampling size is also important regarding the previous data available for this country.

However, the manuscript really needs some improvement to better understand the study, the results and to facilitate the reading.

General point to consider:

- The introduction part of the manuscript doesn’t mention the TES study while it is largely described in the method part.

**Response:** We appreciate the reviewer for this comment. We have now made significant changes to the introduction in the revised manuscript and made mention of the TES study.

- 300 samples have been selected for the study? Two questions: why only 300 out of 586?

**Response:** We only had funding for 300 samples. As a result, we selected 300 samples for molecular analysis.

- And why selected them randomly? If you have some responses to treatment and D3 positivity or not, it would be more interesting to select all the interesting profiles regarding the clinical response than entirely randomly selecting them.

**Response:** Of the 586 patients enrolled, 32 patients had treatment failure across the three states and these samples were selected. After which a random selection of samples across the three states was carried out to make up to 100 samples per state.

- Is this TES has been already published? Why not publish all the results together as they are tightly related?

**Response:** Yes, the TES study has been published. Our current study was centered on molecular surveillance for polymorphisms associated with ART. The TES that was published focused on the clinical responses of patients to the drugs used.

- The abstract mentions in line 27 that the paper “evaluated the status of artemisinin resistance”. It is quite ambitious without in vivo or in vitro correlation. In the best case you evaluate the genomic profile of this gene at the time when Act is used in the country.

**Response:** We appreciate the reviewer for this comment. We have rephrased this statement in the manuscript. Line 29 - 31 in the revised manuscript now reads

“In this study, we evaluated the genomic profile of the *pfk13* gene associated with artemisinin resistance in *Plasmodium falciparum* in Nigerian children, by targeted sequencing of the *pfk13* gene.”

- The part and figure dedicated to the phylogenetic analysis could probably be reduced as less important to answer the question of resistance.

**Response:** We appreciate the reviewer for this comment. We have now removed the section on phylogenetic analysis based on the recommendations of the other two reviewers.

**Minor comments**:

- Introduction line 60 and 346: the information needs to be updated. More mutations are validated by WHO.

**Response:** We appreciate this reviewer for this important observation. The introduction has been updated to include these validated mutations. Line 60 - 63 in the revised manuscript now reads …

“The *pfk13* gene has been reported to be associated with ART-resistance [8, 9]. Several point mutations in the *pfk13* gene (F446I, N458Y, M476I, Y493H, R539T, I543T, P553L, R561H, P574L and C580Y) have been validated to correlate with clinical ART resistance in Southeast Asia and South America [2,10].**”**

- Line 64 suggests that ARTs are the only effective drug to treat P. falciparum. That is not really true so this assessment should be balanced.

**Response:** We appreciate the reviewer for this comment. We have rephrased the statement in the manuscript. Line 64 - 65 in the revised manuscript now reads …

“Due to these mutations, the efficacy of ACTs may be compromised (13).”

- Line 70 mentions that “no systematic molecular epidemiology studies on field …. have so far been conducted”. However, the authors discuss their results according to previous results even if those studies had a lower number of samples. Therefore the “no” should be weight.

**Response:** We appreciate the reviewer for this comment. We have rephrased the statement in the manuscript. Line 70 - 72 in the revised manuscript now reads …

“Despite the need to regularly survey for emergence of these *pfk13* mutant alleles in different malaria-endemic regions of Nigeria, only a small number of systematic molecular epidemiological studies on field *P. falciparum* isolates have so far been conducted [2].”

- Figure 1 legend is missing. This figure could be improved if the malaria transmission area is represented and even more, the intensity of transmission.

**Response:** We appreciate the reviewer for this comment. We have now removed the previous figure 1 from the manuscript due to copyright issues raised by the journal.

- Table 1: for the genotyping aspect, is this table very useful?

**Response:** We appreciate the reviewer for this comment. However, the purpose of Table 1 is to give readers a brief description of the sample demographics for this study.

- Table 2: it would be easier to read if you order the mutation by position in the gene. To have a complete table, it could also be interesting to mention: - the total number of mutant samples find for each mutation and also adding the number of wild type; In this case, figure 2 is no more useful,

**Response:** We appreciate the reviewer for this comment. We have now ordered mutations by position in the gene as suggested. However, we decided to keep figure 2 (now figure 1 in the revised manuscript) because we feel it gives a more interactive view of the percentage of mutations and wild type.

- the TES endpoint at Day 3 and 28 or 42 (mean among the observed cases?)

**Response:** This question is unclear to us. However, if the reviewer is asking if values from these days are means from all samples that had these days of failure, then no they are not.

- if this is the first description of the mutation, if not, are these mutations related with ART R somewhere else.

**Response:** We have reported some novel mutations that have not been reported elsewhere. As such we cannot say if these mutations are associated with ART resistance. Further molecular and *in vivo* assays need to be done to validate the role of these mutations in ART resistance.

- Table 3: as the ∆∆G is important it could be useful to classify them from the less stable to the more stable.

**Response:** We appreciate the reviewer for this comment. We have now removed the protein stability analysis as suggested by the other two reviewers.

- No S5 table in the received document.

**Response:** This was submitted in the original document. This was a table showing K13 mutations in Nigeria (including the ones observed in this study). However, this is now Table 3 in the revised manuscript.

**Table 3: Molecular Surveillance of *Pfk13*** **propeller polymorphisms in Nigeria**

| S/N | Mutations Previously observed | Year observed | Reference |
| --- | --- | --- | --- |
| 1 | G496G | 2014 | [20] |
| 2 | K610R | 2014 | [20] |
| 3 | A626T | 2014 | [20] |
| 4 | A627A | 2014 | [20] |
| 5 | P441S | 2017, 2018 | [21] |
| 6 | C469C* | 2017 | [21] |
| 7 | V589V | 2016 | [21] |
| 8 | V650F | 2016 | [21] |
| 9 | N664N | 2017 | [21] |
| 10 | A676A | 2016 | [21] |
| 11 | Q613H* | 2010, 2011, 2015, 2016 | [22, 23] |
| 12 | K189T | 2015, 2016 | [23] |
| 13 | H136N | 2015, 2016 | [23] |
| 14 | D464N | 2010, 2011 | [22] |
| 15 | G665C | 2016 | [13] |
| 16 | V666V | 2016 | [13] |
| 17 | P553P | 2016 | [13] |
| 18 | V510V | 2016 | [13] |
| 19 | A578S* | 2010, 2011 | [22] |
| 20 | A621A* | 2014 | [24] |
| 21 | K438N | 2018 | Observed in this study |
| 22 | G449S | 2018 | Observed in this study |
| 23 | F451L | 2018 | Observed in this study |
| 24 | N694K | 2018 | Observed in this study |
| 25 | A557S | 2018 | Observed in this study |
| 26 | V487E | 2018 | Observed in this study |
| 27 | V692G | 2018 | Observed in this study |
| 28 | Q661H | 2018 | Observed in this study |
| 29 | N664I | 2018 | Observed in this study |

***Mutations observed in previous studies and our study**
