# Supplementary material for: Molecular profiling of the artemisinin resistance Kelch 13 gene in Plasmodium falciparum from Nigeria
Source: PLoS One. 2022 Feb 28;17(2):e0264548. doi: 10.1371/journal.pone.0264548 (PMC8884509; doi:10.1371/journal.pone.0264548)
Supplement: Attachment — Submitted filename: Response to Editor.docx [file pone.0264548.s003.docx]

Comments/suggested changes from the editor:

**General**:

Please use abbreviations consistently throughout the manuscript and write them in full the first time they appear in the manuscript. There are also a couple of typos and some grammar errors which should be corrected.

**Response:** We appreciate the editor for your thorough review of our manuscript. We have taken note of your comments and made the necessary corrections.

**Title**:

1. Change to “Molecular profiling of the artemisinin resistance Kelch 13 gene in *Plasmodium falciparum* from Nigeria”

**Response:** We appreciate the editor for your comment. We have now changed the title of the manuscript to “Molecular profiling of the artemisinin resistance Kelch 13 gene in *Plasmodium falciparum* from Nigeria” as suggested. Line 1 - 2 in the revised manuscript now reads …

“**Full Title: Molecular profiling of the artemisinin resistance Kelch 13 gene in *Plasmodium falciparum* from Nigeria”**

 Introduction:

2. Line 57: Delete “The” before “Artemisinin-resistant”.

**Response:** We have removed ‘The’ from the statement. Line 57 in the revised manuscript now reads …

“...Artemisinin (ART)-resistant *P. falciparum* has been…”

3. Line 59: Also cite doi: 10.1056/NEJMc0805011 alongside REF #3

**Response:** This has now been cited in the manuscript. Line 59 in the revised manuscript now reads …

“border [3, 4] and has spread to other malaria endemic regions [5 – 8].

4. Line 60: The first sentence should be deleted.

**Response:** This sentence has been deleted. Line 60 in the revised manuscript now reads …

“Several point mutations in the *pfk13* gene (F446I, N458Y, M476I, Y493H, R539T, I543T,”

5. Line 64: Cite REF #8 alongside REFs #11 and #12.

**Response:** We thank the editor for your comment. Line 63 in the revised manuscript now reads …

“...survival rates based on Ring-stage Survival Assays (RSA)0–3 h [10-12]*.*”

6. Lines 64-65: “Due to these mutations, the efficacy of ACTs may be compromised”. REF #13 is inappropriate. Furthermore, I would expect some lines about partner drug resistance here. See also comment of Reviewer #1.

**Response:** We appreciate the editor for your comment. We have now changed the reference in the revised manuscript and also added a few statements on partner drug resistance. Line 63 - 68 in the revised manuscript now reads …

“Furthermore, mutations and increased copy numbers of genes such as *P. falciparum* multidrug resistant gene-1 (*pfmdr1*) and *P. falciparum* chloroquine resistance transporter (*pfcrt*) gene have been linked to resistance in artemisinin (and derivatives) partner drugs such as lumefantrine, amodiaquine, mefloquine and piperaquine [13-18]. Due to these mutations, the efficacy of ACTs may be compromised [19].”

7. Line 69: Delete REF #15 (Colombian study).

**Response:** This has been deleted. Line 73 in the revised manuscript now reads …

“no association between ART-resistant parasites and the ten validated mutations has been found [20-23].”

**Materials and Methods:**

8. Line 141: iii) Asexual parasite clearance time (PCT) to line 142. I also suggest including the commonly used terms ACPR, ETF, LCF, and LPF, respectively, as used in the final TES report. These would not have to be described/defined in detail in the manuscript; the official WHO TES study protocol can be cited instead.

**Response:** We appreciate the editor for your comment. We have now included the ACPR, ETF, LCF and LPF as you suggested. Line 142 - 155 in the revised manuscript now reads …

“Response to drug treatment was evaluated using the following treatment indices:

(i)   Adequate clinical and parasitological response (ACPR), early treatment failure (ETF), late clinical failure (LCF) and late parasitological failure (LPF) [Full description of WHO TES study protocol available at https://www.who.int/docs/default-source/documents/publications/gmp/methods-for-surveillance-of-antimalarial-drug-efficacy.pdf?sfvrsn=29076702_2].

(ii)    Asexual parasite reduction ratio (PRR) one or two days after treatment initiation (PRRD1 or PRRD2) defined as the ratio of asexual parasitaemia pre-treatment initiation and that on day one or two, respectively.

(iii)   Asexual parasite positivity on day 1 (APPD1), 2 (APPD2) or 3 (APPD3) defined as proportion of children with residual asexual parasitaemia one, two or three days after treatment initiation, respectively Asexual parasite clearance time (PCT) defined as the time elapsing between drug administration and absence of microscopic detection of viable asexual parasitaemia.”

9. Lines 145-146: This sentence needs to be reworded.

**Response:** We have now reworded this statement in the manuscript. Line 159 - 161 in the revised manuscript now reads …

“A total of 300 pre-treatment (Day 0) DBS filter paper samples from all three sites were utilised in this study. Of the 300 samples, 32 had LPF making a final total of 332 samples selected for analysis.”

**Results**:

10. Between “Demographics” and *Pfk13* gene mutations”, a short summary of the TES results (i.e., summary table of crude and PCR-corrected ACPR, ETF, LCF, and LPF) of the 300 samples would be very helpful.

**Response:** We appreciate the editor for your comment. We have now included a table describing the summary of the TES study as Table 2. Table 2 in the revised manuscript now reads …

“**Table 2: Summary of treatment outcome by State and drug**

| **State** | **Treatment**  **Outcome** | **Drug**    **AA                    AL                 DHP** | **Total** |
| --- | --- | --- | --- |
| **Enugu** | **Number of samples**  ETF  LCF  LPF  ACPR_u  ACPR_c  %ACPR_c | **50**                      **50**                             0                        0                             0                        0                            11                       2                            39                      48                            49                      50                            98                     100 | **100**  0  0  14  87  99  99 |
| **Kano** | **Number of samples**  ETF  LCF  LPF  ACPR_u  ACPR_c  %ACPR_c | **50                      50**   0                        0   0                        0   2                        9  48                      41  50                      50  100                   100 | **100**  0  0  11  89  100  100 |
| **Plateau** | **Number of samples**  ETF  LCF  LPF  ACPR_u  ACPR_c  %ACPR_c | **50                      50**   0                        0   0                        0   1                        7  49                      43  50                      50  100                   100 | **100**  0  0  8  92  100  100 |
| **Total** | **Number of samples**  ETF  LCF  LPF  ACPR_u  ACPR_c  %ACPR_c | **100**                 **150                      50**   0                        0                          0   0                        0                          0   3                       27                         2  97                     123                      47  100                   149                      50  100                   99.3                   100 | **300**  0  0  32  267  299  99.7 |

**Crude ACPR (ACPR_u); PCR-corrected ACPR (ACPR_c)**

**WHO protocol for parasite genotyping to differentiate recrudescence from new infections available at http://whqlibdoc.who.int/publications/2008/9789241596305_eng.pdf**

11. Lines 222-224: Replace with: “Thirteen pfk13 gene mutations were detected in 21 out of the 332 sequences analyzed in this study (Table 2).”

**Response:** We have effected this change in the manuscript. Line 240 - 241 in the revised manuscript now reads …

“...Thirteen *pfk13* gene mutations were detected in 21 out of the 332 sequences analysed in this study (Table 3)...”

12. Line 225: Delete “and”.

**Response:** This has been deleted. Line 242 in the revised manuscript now reads …

“chromosome 13 in the propeller region of the *pfk13* protein was 6.3%, 93.7% did not have…”

13. In Figure 1 and Table 3, please order the SNPs in ascending order according to the location in the gene.

**Response:** We appreciate the editor for your comment. We have now ordered the SNPs in ascending order according to the location in the gene in Figure 1 and Table 3 (now Table 4 in the revised manuscript). Figure 1 and Table 4 in the revised manuscript now reads thus …

**Fig 1. A chart showing the frequency distribution of *pfk13* gene polymorphisms observed in this study**

**Table 4: Molecular Surveillance of *Pfk13*** **propeller polymorphisms in Nigeria**

| S/N | Mutations Previously observed | Year observed | Reference |
| --- | --- | --- | --- |
| 1 | H136N | 2015, 2016 | [26] |
| 2 | K189T | 2015, 2016 | [26] |
| 3 | E433G | 2018 | [27] |
| 4 | F434I | 2018 | [27] |
| 5 | F434S | 2018 | [27] |
| 6 | K438N | 2018 | Observed in this study |
| 7 | P441S | 2017, 2018 | [28] |
| 8 | F442F | 2018 | [27] |
| 9 | G449S | 2018 | Observed in this study |
| 10 | F451L | 2018 | Observed in this study |
| 11 | D464N | 2010, 2011 | [29] |
| 12 | C469C* | 2017 | [28] |
| 13 | V487E | 2018 | Observed in this study |
| 14 | F492F | 2018 | [27] |
| 15 | G496G | 2014 | [30] |
| 16 | V510V | 2016 | [20] |
| 17 | P553P | 2016 | [20] |
| 18 | A557S | 2018 | Observed in this study |
| 19 | A578S* | 2010, 2011 | [29] |
| 20 | V589V | 2016 | [28] |
| 21 | K610R | 2014 | [30] |
| 22 | Q613H* | 2010, 2011, 2015, 2016 | [29, 26] |
| 23 | A621A* | 2014 | [31] |
| 24 | A626T | 2014 | [30] |
| 25 | A627A | 2014 | [30] |
| 26 | V650F | 2016 | [28] |
| 27 | Q661H | 2018 | Observed in this study |
| 28 | N664I | 2018 | Observed in this study |
| 29 | N664N | 2017 | [28] |
| 30 | G665C | 2016 | [20] |
| 31 | V666V | 2016 | [20] |
| 32 | A676A | 2016 | [28] |
| 33 | I684N | 2018 | [27] |
| 34 | I684T | 2018 | [27] |
| 35 | E688K | 2018 | [27] |
| 36 | V692G | 2018 | Observed in this study |
| 37 | N694K | 2018 | Observed in this study |

***Mutations observed in previous studies and our study**

14. Lines 226 and 227: Replace “sequences” with “samples”.

**Response:** This change has been effected in the manuscript. Line 243 - 245 in the revised manuscript now reads …

“The highest occurring mutation Q613H (1.5%) was detected in five samples (three from Plateau State and two from Enugu State). The C469C mutation with 1.2% was detected in four samples from Enugu State.”

15. Table 3 was referred to in line 224. However, this is not correct because the text outlined the results of the current study. Table 3 summarises the results of all Nigerian studies hitherto conducted; this should be mentioned in the text. On another note: Is there a specific reason why the polymorphisms reported by Abubakar *et al*. (2020; DOI:10.3390/tropicalmed5020085) are not included in Table 3?

**Response:** We appreciate the editor for this comment. We have now correctly referred to Table 3 (now Table 4 in the revised manuscript) in the manuscript. There was no particular reason why we didn’t report the polymorphisms reported by Abubakar *et al.* , it was an omission on our part. However, we have now included the polymorphisms in our table 4. Line 246 - 247 in the revised manuscript now reads …

“…We summarized mutations detected in this study and previous Nigerian studies in Table 4.”

**Table 4: Molecular Surveillance of *Pfk13*** **propeller polymorphisms in Nigeria**

| S/N | Mutations Previously observed | Year observed | Reference |
| --- | --- | --- | --- |
| 1 | H136N | 2015, 2016 | [26] |
| 2 | K189T | 2015, 2016 | [26] |
| 3 | E433G | 2018 | [27] |
| 4 | F434I | 2018 | [27] |
| 5 | F434S | 2018 | [27] |
| 6 | K438N | 2018 | Observed in this study |
| 7 | P441S | 2017, 2018 | [28] |
| 8 | F442F | 2018 | [27] |
| 9 | G449S | 2018 | Observed in this study |
| 10 | F451L | 2018 | Observed in this study |
| 11 | D464N | 2010, 2011 | [29] |
| 12 | C469C* | 2017 | [28] |
| 13 | V487E | 2018 | Observed in this study |
| 14 | F492F | 2018 | [27] |
| 15 | G496G | 2014 | [30] |
| 16 | V510V | 2016 | [20] |
| 17 | P553P | 2016 | [20] |
| 18 | A557S | 2018 | Observed in this study |
| 19 | A578S* | 2010, 2011 | [29] |
| 20 | V589V | 2016 | [28] |
| 21 | K610R | 2014 | [30] |
| 22 | Q613H* | 2010, 2011, 2015, 2016 | [29, 26] |
| 23 | A621A* | 2014 | [31] |
| 24 | A626T | 2014 | [30] |
| 25 | A627A | 2014 | [30] |
| 26 | V650F | 2016 | [28] |
| 27 | Q661H | 2018 | Observed in this study |
| 28 | N664I | 2018 | Observed in this study |
| 29 | N664N | 2017 | [28] |
| 30 | G665C | 2016 | [20] |
| 31 | V666V | 2016 | [20] |
| 32 | A676A | 2016 | [28] |
| 33 | I684N | 2018 | [27] |
| 34 | I684T | 2018 | [27] |
| 35 | E688K | 2018 | [27] |
| 36 | V692G | 2018 | Observed in this study |
| 37 | N694K | 2018 | Observed in this study |

***Mutations observed in previous studies and our study**

16. Line 251: “...(mean time to recurrence: 29±6.3 days).”

**Response:** This has been corrected in the manuscript. Line 279 - 280  in the revised manuscript now reads …

“within 21-42 days of follow-up post treatment initiation (mean time to recurrence: 29±6.3 days),…”

17. Footnote Table 4/Line 263: “...recurrent parasitaemia”

**Response:** This has been corrected in the manuscript. Line 286 in the revised manuscript now reads …

“ ***Geometric mean; ^#^Mean LPF;^+^Only sample with recrudescence recurrent parasitaemia”**

18. Lines 266-267: Replace with “Comparison of responsiveness indices following treatment

initiation in children with and without mutated *Pfk13*.”

**Response:** We appreciate the editor for your comment. This has now been corrected in the manuscript. Line 290 - 291 in the revised manuscript now reads …

“**Comparison of responsiveness indices following treatment initiation in children with and without mutated *Pfk13*”**


**Discussion**:

19. The first sentence of the Discussion should be toned down, particularly in view of the fact that no SNPs associated with artemisinin resistance were observed and nothing is reported on partner drug resistance.

**Response:** We appreciate the editor for this comment. We have now rephrased this statement in the manuscript. Line 303 - 305 in the revised manuscript now reads ..

“The knowledge of mutations in the *pfk13* gene associated with slow clearance of artemisinin derivatives provides the ability to track the emergence and prevent the spread of resistant parasites and assess the effectiveness of control measures.”

20. Line 300: Replace “is” with “was”.

**Response:** This has been changed in the manuscript. Line 307 in the revised manuscript now reads …

“...None of them was among the ten”

21. Line 317: Replace “has” with “have”.

**Response:** This has been changed in the manuscript. Line 323 in the revised manuscript now reads …

“of our knowledge, have not yet been described elsewhere…”

22. Line 322: “...are less likely to be associated with a delayed parasite clearance phenotype...”

**Response:** This has been changed in the manuscript. Line 328 in the revised manuscript now reads …

“are less likely to be associated with a delayed parasite clearance phenotype as evidenced by”

23. Lines 324-326: “In addition, significantly longer asexual parasite clearance times in children infected with non-mutant *Pfk*13 parasites indicate that mutants identified in the parasites circulating in Nigeria do not confer resistance to artemisinin derivatives.

**Response:** This has been changed in the manuscript. Line 330 - 332 in the revised manuscript now reads …

“In addition, significantly longer asexual PCT in children infected with non-mutant *pfk13* parasites indicate the mutants identified in the parasites circulating in Nigeria do not confer resistance to artemisinin derivatives.”

**Supporting Information**:

24. The Supporting Information could be omitted. Instead, a reference to the WHO protocol for parasite genotyping to differentiate recrudescence from new infections could be in included in the footnote of the summary table I suggested in comment # 10.

**Response:** We appreciate the editor for your comment. The supporting information has now been removed and a reference to the WHO protocol has been included as a footnote to Table 2 as suggested. Table 2 in the revised manuscript now reads thus …

**Table 2: Summary of treatment outcome by State and drug**

| **State** | **Treatment**  **Outcome** | **Drug**    **AA                    AL                 DHP** | **Total** |
| --- | --- | --- | --- |
| **Enugu** | **Number of samples**  ETF  LCF  LPF  ACPR_u  ACPR_c  %ACPR_c | **50**                      **50**                             0                        0                             0                        0                            11                       2                            39                      48                            49                      50                            98                     100 | **100**  0  0  14  87  99  99 |
| **Kano** | **Number of samples**  ETF  LCF  LPF  ACPR_u  ACPR_c  %ACPR_c | **50                      50**   0                        0   0                        0   2                        9  48                      41  50                      50  100                   100 | **100**  0  0  11  89  100  100 |
| **Plateau** | **Number of samples**  ETF  LCF  LPF  ACPR_u  ACPR_c  %ACPR_c | **50                      50**   0                        0   0                        0   1                        7  49                      43  50                      50  100                   100 | **100**  0  0  8  92  100  100 |
| **Total** | **Number of samples**  ETF  LCF  LPF  ACPR_u  ACPR_c  %ACPR_c | **100**                 **150                      50**   0                        0                          0   0                        0                          0   3                       27                         2  97                     123                      47  100                   149                      50  100                   99.3                   100 | **300**  0  0  32  267  299  99.7 |

**Crude ACPR (ACPR_u); PCR-corrected ACPR (ACPR_c)**

**WHO protocol for parasite genotyping to differentiate recrudescence from new infections available at http://whqlibdoc.who.int/publications/2008/9789241596305_eng.pdf**
